# Improving Cleaning and Disinfection of High-Touch Surfaces in Intensive Care during Carbapenem-Resistant *Acinetobacter baumannii* Endemo-Epidemic Situations

**DOI:** 10.3390/ijerph15102305

**Published:** 2018-10-19

**Authors:** Beatrice Casini, Anna Righi, Nunzio De Feo, Michele Totaro, Serena Giorgi, Lavinia Zezza, Paola Valentini, Enrico Tagliaferri, Anna Laura Costa, Simona Barnini, Angelo Baggiani, Pietro Luigi Lopalco, Paolo Malacarne, Gaetano Pierpaolo Privitera

**Affiliations:** 1 Department of Translational Research, N.T.M.S., University of Pisa, 56123 Pisa, Italy; righianna@gmail.com (A.R.); micheleto@hotmail.it (M.T.); giorgiserena@yahoo.it (S.G.); laviniazezza@alice.it (L.Z.); paola.valentini@dps.unipi.it (P.V.); alauracosta@alice.it (A.L.C.); angelo.baggiani@med.unipi.it (A.B.); pierluigi.lopalco@unipi.it (P.L.L.); gaetano.privitera@med.unipi.it (G.P.P.); 2 Anesthesia and Intensive Care Unit PS, University Hospital, 56124 Pisa, Italy; n.defeo@med.unipi.it (N.D.F.); pmalacarne@hotmail.com (P.M.); 3 Infectious Disease Unit, University Hospital, 56124 Pisa, Italy; tagliaferrienrico@alice.it; 4 Unit of Microbiology, Azienda Ospedaliero Universitaria Pisana, 56124 Pisa, Italy; s.barnini@ao-pisa.toscana.it

**Keywords:** high-touch surfaces, carbapenem-resistant *A. baumannii*, pre-impregnated wipes, outsourced cleaning services

## Abstract

*Aims:* High-touch surfaces cleaning and disinfection require the adoption of effective and proper executed protocols, especially during carbapenem-resistant *Acinetobacter baumannii* (CRAB) endemo-epidemic situations. We evaluated the effectiveness and residual disinfectant activity of disposable pre-impregnated wipes (Modified Operative Protocol, MOP) in reducing environmental bioburden versus a two-step Standard Operative Protocol (SOP) in a 12-bed Intensive Care Unit. *Methods:* Five high-touch surfaces were cleaned and disinfected either according to the SOP (alcohol-based cleaning and chlorine-based disinfection) or using quaternary ammonium compounds-based disposable wipes (MOP). Sampling was performed before each procedure and at 0.5, 2.5, 4.5 and 6.5 h after (560 sites). Total viable count (TVC) was evaluated according to Italian hygiene standard (<50 CFU/24 cm^2^). Clinical and environmental CRAB strains isolated were genotyped. *Results:* On non-electromedical surfaces the difference between TVC before procedure and at each of the following times was significant only for the MOP (*p* < 0.05, Wilcoxon test). Using the MOP, only 7.4% (10/135) of sites showed TVC >50 CFU/24 cm^2^ (hygiene failures) versus 18.9% (25/132) after SOP (*p* < 0.05, Fisher’s Exact test). On infusion pumps a higher number of hygiene failures was observed after the SOP (7/44, 15.9%) compared with the MOP (4/45, 8.9%). Genotyping highlighted a common source of infection. *Conclusion:* On high-touch surfaces, the use of disposable wipes by in-house auxiliary nurses may represent a more effective alternative to standard cleaning and disinfection procedure performed by outsourced cleaning services, showing effectiveness in reducing microbial contamination and residual disinfection activity up to 6.5 h.

## 1. Introduction

High-touch surfaces are recognized as a possible reservoir of infectious agents and their contamination can pose a risk also for the spread of multi-resistant organisms [1,2,3,4], hence they are recommended to be cleaned and disinfected on a more frequent schedule than minimal touch surfaces [5]. Environmental cleaning and disinfection (C&D) are important components of a comprehensive strategy in order to control healthcare-associated infections [6,7,8,9], especially in wards such as Intensive Care Unit (ICU) where patients are compromised. Studies evaluating improved cleaning interventions have reported that approximately 5–30% of surfaces remain potentially contaminated due to the inability of existing detergent and disinfectants formulations to disrupt biofilms [10].

To achieve higher rates of effectiveness in the field, new C&D strategies should be evaluated. The ready-to-use wipes are increasingly used in health care settings, although different antimicrobial wipes have shown a variable effectiveness in removing microbial bioburden from inanimate surfaces and in reducing the pathogens transfer between surfaces [11]. As reported by Sattar et al. [12], the use of wipes containing 0.5% accelerated H_2_O_2_ or sodium hypochlorite solution <3% were effective in removing both *Acinetbacter baumannii* and *Staphylococcus aureus* (at least 7 log_10_ CFU reduction). Kenters et al., evaluated the effectiveness of different cleaning-disinfectant wipes with different composition demonstrating for all the type of wipes a log_10_ reduction higher than 5 with a 5-min exposure time on *Klebsiella pneumoniae* OXA-48, *A. baumannii* and VRE outbreak strains [13].

The aim of this study was to evaluate the effectiveness on the field of pre-impregnated wipes (Modified Operative Protocol, MOP) to reduce environmental bacterial burden and to maintain a disinfection activity on high-touch surfaces as an alternative to the currently used Standard Operative Protocol (SOP) in a 12-bed ICU during a carbapenem-resistant *A. baumannii* (CRAB) endemo-epidemic situation.

## 2. Materials and Methods

*Setting*: The study was performed in a 12-bed ICU at a university Italian hospital. During the study, from 1 March to 30 April 2016, 82 patients (mean age 66.7 ± 16.4 years) were admitted to the ICU and eight were already hospitalized (case mix: 21 trauma, 14 emergency surgery, eight elective surgery, 47 general medicine), 41 of which ventilated for at least 24 h. The bed turnaround was 6.6 and the average occupancy 88%.

In this hospital, cleaning services was outsourced and according to the SOP, housekeeping staff using disposable clothes, applied an alcohol-based detergent (Keradet, Kiehl, Odelzhausen, Germany) followed by a chlorine-based disinfectant (Antisapril 2%, Angelini, Rome, Italy, active chlorine 540 mg/L) on furniture surfaces except electromedical devices when in use. Monitors and pumps were sanitized only at the patient discharge.

During the study, according to the MOP, on two units disposable wipes impregnate with cationic surfactant tensioactives, quaternary ammonium compounds and biguanide (Clinell Universal Wipes, GAMA, Watford, UK) were applied by in-house auxiliary nurses, trained about the proper use of wipes. According to manufacturer’s instructions, a “one wipe, one surface, one direction” approach was adopted. The two units treated with the MOP were compared with two of the units managed with the SOP.

**Figure 1:**
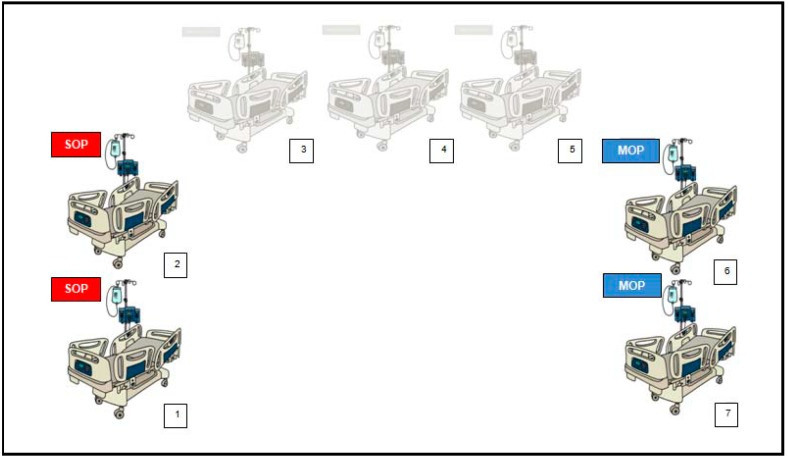
Patient units selected for the study (2 in each opposite side of the open-space, functionally separated) and cleaning and disinfection procedures applied in each units (SOP: Standard Operative Protocol, MOP: Modified Operative Protocol).

*Sampling procedure*: Five inanimate surfaces for each patient unit were chosen to determine the bacterial bioburden before and after both the SOP and the MOP. In Figure 1, the patient units selected and include in the study and C&D procedures applied in each unit are reported. In order to evaluate the temporal trend of microbial contamination [14,15], samples were obtained immediately before and at 0.5, 2.5, 4.5 and 6.5 h after each C&D procedure (overall 560 samples).

According to ISO 14698-1, 55-mm diameter Rodac plates containing Plate Count Agar, PCA, with neutralizers (VWR International PBI, Radnor, PA, USA) were used for TVC enumeration and Violet Red Bile Dextrose Agar, VRBD, (Oxoid, Basingstoke, UK) for Gram-negative bacteria qualitative evaluation. Contact plates were incubated aerobically at 37 °C for 48 h.

Suspect *Acinetobacter* spp. or *Klebsiella* spp. were subcultured on chromID™ mSuperCARBA (bioMérieux, Marcy l’Etoile, France) and identified by API/ID32 Strep Miniature System (bioMérieux).

The total microbial load and the presence of pathogens were evaluated according to the hygienic standards proposed by the Italian National Guidelines (for the ICU: <50 CFU/plate -24 cm^2^- and absence of pathogens) [16].

In the ICU, a systematic surveillance for CRAB colonization/infection was performed through weekly rectal swabs and/or bronchial aspirate sampling. Clinical and environmental CRAB strains were genotyped in order to assess the source of nosocomial colonization/infection, comparing the genomic profile according to the PFGE Typing Protocol recommended for *A. baumannii* [17,18].

*Statistical analysis*: For each C&D procedure, we compared TVC at baseline and at each of the following times using Wilcoxon rank-sum test and the number of hygiene failures (environmental samples with TVC > 50 CFU/24 cm^2^) using McNemar’s test with continuity correction. We compared the number of hygiene failures between the two protocols with the Fisher’s Exact test. (Epi Info version 7.2, CDC, GA, USA).

## 3. Results

After 0.5 h from the C&D, the initial average TVC detected on all non-electromedical high-touch surfaces (bed rails, overbed table, worktop) was reduced from 34 CFU/24 cm^2^ (SD ± 44 CFU/24 cm^2^) to 21 CFU/24 cm^2^ (SD ± 31 CFU/24 cm^2^) after the SOP, and from 52 CFU/24 cm^2^ (SD ± 63 CFU/24 cm^2^) to 15 CFU/24 cm^2^ (SD ± 24 CFU/24 cm^2^) after the MOP. The percentage decrease was −38.2% (Wilcoxon test, *p* = 0.3192) and −71.2%, （Wilcoxon test, *p* = 0.0005) respectively.

**Figure 2:**
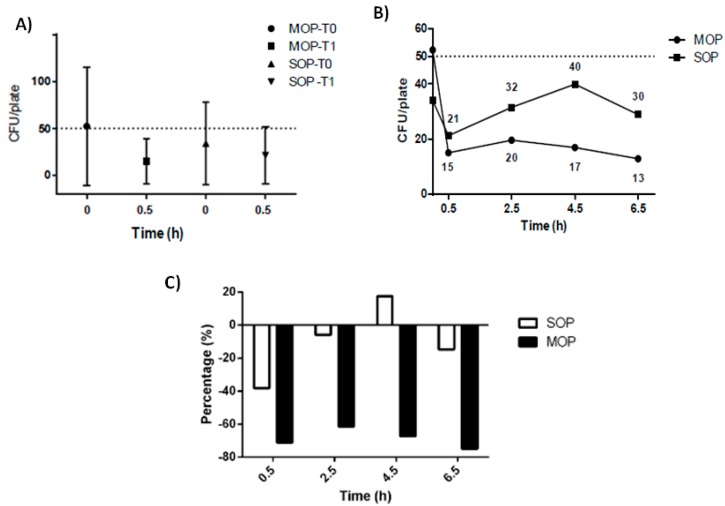
Trend of the Total Viable Counts (TVCs) and percentage reduction of the values before and after the application of the cleaning and disinfection procedures on non-electromedical surfaces: (**A**) Average TVCs before and after 0.5 h the two procedures (**B**) Temporal trend of TVCs during the 6.5 h of sampling (**C**) Percentage reductions of the TVCs. Note: The dashed line represents the targeted bacterial burden proposed by the Italian Workers Compensation Authority (TVC < 50 CFU/plate, plate equal to 24 cm^2^).

In Figure 2, the Total Viable Counts (TVCs) trend and the percentage reduction of the values before and after the application of the two different procedures on non-electromedical surfaces are reported.

According to the hygienic standard proposed by the national guidelines (TCV < 50 CFU/24 cm^2^), before the procedures a comparable hygienic level on all the surfaces was observed, since no significant differences resulted between the hygiene failures found on surfaces subsequently treated with the SOP (9/33 failures) and those dealt with the MOP (13/36) (*p* = 0.60, Fisher’s Exact test).

After the SOP on non-electromedical surfaces, hygiene failures were 25 out of 132 (18.9%) versus 10 out of 135 after the MOP (7.4%), (*p* < 0.05, Fisher’s Exact test). In Table the hygiene failures and the pathogens found on each type of high-touch surface are reported.

Similarly, the difference between the number of the hygiene failures immediately before the procedure and at each of the subsequent times was statistical significant only for the MOP (*p* < 0.05, McNemar’s test with continuity correction).

Considering only sites with hygiene failures at baseline, using the MOP 11/13 surfaces (84.6%) met the hygienic standard at all the following times, while applying the SOP only 3/9 (33.3%) (*p* < 0.05, Fisher’s Exact test). The ability to preserve clean surfaces with basal TVC < 50 CFU/24 cm^2^ was greater for the MOP but not significantly (13/20 for the wipe protocol, 11/24 for the SOP).

On monitors, TVCs resulted <50 CFU/24 cm^2^ in almost all (111/112) of the sampled sites and no pathogens were isolated. On pumps under the MOP only the TVC at 2.5 h was significantly lower than the baseline one (Wilcoxon test), while under the SOP, the TVCs at 2.5 h and at 6.5 h were significantly higher than the basal ones. The McNemar’s test with continuity correction pointed out no significant difference either for the MOP or for the SOP (Table 1).

Since there was no significant difference between the hygiene failures before the procedure (1/12 for the MOP, 0/11 for the SOP), we compared the total hygiene failures at the subsequent times performing the Fisher’s Exact test: the hygiene failures were higher (not significantly) for the SOP: 4/45 (8.9%) for the MOP and 7/44 (15.9%) for the SOP. Considering only the hygiene failures at 6.5 h, the difference between the two protocols was significant (0/11 for the MOP versus 5/11 for the SOP).

### Carbapenem-Resistant A. baumannii Strains

During the studied period, four patients resulted colonized/infected with CRAB, one of which in contact precautions in a single room due to sepsis and respiratory-gastrointestinal colonization by CRAB as well as respiratory-gastrointestinal colonization by KPC-producing *K. pneumoniae*. The single room was included only once in the monitoring to increase the recovery of CRAB from surfaces. Overall 23 on 100 environmental samples were positive (23%) in the high-touch area surrounding three of the four patients (Table 1). In particular, in the single room, 15/25 high-touch surfaces resulted positive for CRAB, but none for *K. pneumoniae.* No statistically significant differences were found between the different protocols and considering the temporal trend of each of them. The clinical CRAB strains were genetically similar to the environmental ones (similarity >95%), suggesting that the clonal spread of CRAB in the ICU played an important role in the endemic/epidemic situation.

## 4. Discussion

In order to assess the environmental hygiene level in hospital, as visual cleanliness does not always correlate with microbiological cleanliness, and in the presence of microbial contamination, microbiological limit considered safe on high-touch sites are not always standardized. Dancer et al., proposed as hygiene standards, <1 CFU/cm^2^ for pathogens and a <5 CFU/cm^2^ as a starting point and <2.5 CFU/cm^2^ as a more suitable goal although with no distinction between different care settings (low-risk or high-risk patients) [19]. These standards were aimed at hospitals using detergent cleaning and double sided dipslides for sampling. For intensive care units the Italian Workers Compensation Authority [16] suggests the use of contact plate and proposes as standard <50 CFU/plate (24 cm^2^) and the absence of pathogens (*S. aureus*, *Pseudomonas aeruginosa*, enterobacteria, *Aspergillus* spp.).

To clean and disinfect environmental surfaces that necessitate low-level disinfection, the housekeeping staff traditionally uses a two-step method (detergent and subsequent disinfectant) with disposable or reusable cloths. To avoid contamination spread, reusable cloths should be adequately cleaned and disinfected [3]. Disposable cloths could solve this potential weakness.

Pre-impregnated disposable cloths cleaning and disinfecting in one step could make the sanitization process faster and easier with consequent increase in cleaning staff compliance, although environmental impact due to waste disposal and cost-effectiveness should be considered [20]. Furthermore, since wipes showed their efficacy to remove and retain Gram negative bacteria, they might be an appropriate choice in health-care settings where CRAB is endemic [11,12,13].

In our study, only the pre-impregnated wipe protocol was effective in reducing significantly TVC or hygiene failures on non-electromedical surfaces from baseline up to 6.5 h, showing a long residual disinfection activity and a better performance when compared to the standard operative protocol, especially when the surfaces were highly contaminated (TVC > 50 CFU/24 cm^2^).

On electromedical-devices, such as the monitors, although not routinely sanitized, almost all TVC values were compliant with the standard and no pathogens were isolated. However, on infusion pumps was observed a higher number of hygiene failures after the SOP, although the difference between the two protocols was not as evident as on non-electromedical surfaces, probably because of the fewer sampled sites. Even if not always significant, data showed a greater effectiveness of pre-impregnated wipes compared to the SOP. It is known that the physical structure of the wipe influences the capacity to pick up and hold soils, microbes, and particles [21], for this reason the higher proportion of removal demonstrated by the wipes protocol compared to the SOP may be due to physical effect alone of the material of which the wipes are made.

The recovery on environmental surfaces (five isolated on the infusion pumps, four on the worktops and two on the bedrails), of CRAB strains genetically similar to clinical strains suggested the importance of high-touch sites as a source of nosocomial colonization/infection, stressing the importance of an appropriate high-touch near-patient sites C&D.

### Our Study has Some Limitations

Firstly, we did not supervise the housekeeping staff who performed the SOP, but this activity was conducted by the hospital executive director of the contract. We do not really know if they strictly complied with the terms of the contract, even though no penalty was applied. Housekeepers generally receive little or no training [8]; this lack of competence can lead to inadequate environmental cleaning, regardless of the products in use. Moreover, outsourced cleaning services are associated with worse patient perception of cleanliness [22]. On the other hand, in-house auxiliary nurses were trained about the proper use of wipes. Secondly, evaluating the CRAB-surface contamination, we did not observe a reduction of the CRAB-positive sites, probably due to the small size of data. Sattar et al., showed a >5 log_10_ reduction in *A. baumannii* applying wipes with similar active ingredients [23]. The effectiveness of these wipe against CRAB should be further investigated.

## 5. Conclusions

Hospitals are encouraged to develop programs to optimize the thoroughness of high-touch surface cleaning in high-risk areas, especially when CRAB is epidemic or endemic as strongly recommended by WHO [9]. However is very difficult to implement cleaning and disinfection strategies when there is extensive outsourcing of hospital cleaning services. Contracted-out services are considered too inflexible to deal with changing circumstances, including unscheduled cleaning or changes in products and protocols. The use of disposable wipes by in-house auxiliary nurses on near-patient inanimate surfaces may represent a more effective alternative to the two-step procedures performed by outsourced cleaning services in reducing the microbial contamination. Auxiliary nurses have a greater awareness of the crucial rule of cleaning and disinfection in infection prevention and they could be trained about the proper use of wipes. Few studies investigated the rate of recontamination over time on frequently touched sites after use of cleaning wipes [14] or disinfectants [15,23] and only one [24] evaluates the temporal trend specifically using wipes designed for one-step C&D as performed in our study.

## Figures and Tables

**Table 1:** Hygiene failures before and after the cleaning and disinfection procedures on non-electromedical surfaces and on the infusion pumps. Note: t0 = before cleaning and disinfection, t1 = 30 min after, t2 = 2.5 h after, t3 = 4.5 h after, t4 = 6.5 h after. Grey boxes indicate the hygiene failures (>50 CFU/24 cm^2^); ***Bed*** in bold italics indicates a bed occupied by a CRAB positive patient; the hash symbol (#) indicates environmental site positive for CRAB and the asterisk symbol (*) indicates patient transferred to another ward.

	**Wipe Protocol**																																			
**Times**	**Bedrails**												**Overbed Table**												**Worktop**											
	Day 1		Day 2		Day 3		Day 4			Day 5			Day 1		Day 2		Day 3		Day 4			Day 5			Day 1		Day 2		Day 3		Day 4			Day 5		
	Bed 1	Bed 2	Bed 1	Bed 2	Bed 1	Bed 2	Bed 1	Bed 2	***Bed 3***	Bed 1	Bed 2	***Bed 3***	Bed 1	Bed 2	Bed 1	Bed 2	Bed 1	Bed 2	Bed 1	Bed 2	***Bed 3***	Bed 1	Bed 2	***Bed 3***	Bed 1	Bed 2	Bed1	Bed 2	Bed1	Bed 2	Bed 1	Bed 2	***Bed 3***	Bed 1	Bed 2	***Bed 3***
t0	13	11	33	68	0	1	18	9	23	0	3	165 #	9	45	1	4	0	1	108	63	64	0	6	259 #	22	37	159	16	126	19	99	137	96	38	70	161 #
t1	0	0	0	16	0	5	12	52	0	0	0	20 #	0	7	0	6	29	2	10	4	0	0	84	9 #	102	38	59	6	4	3	3	4	11	6	22	29 #
t2	3	3	0	19	1	*	5	10	2	4	8	1 #	3	15	11	1	3	*	25	24	0	0	31	21 #	1	176	39	13	106	*	4	24	0	55	0	40
t3	103	73	9	6	31	*	7	22	9	0	18	44 #	9	17	3	0	3	*	6	3	0	1	39	44 #	1	23	9	14	8	*	13	3	0	6	9	27 #
t4	88	23	0	0	0	*	2	7	2	22	3	34	0	9	0	1	0	*	3	4	1	0	1	34 #	26	18	28	6	8	*	29	16	10	3	8	41
	**Standard Protocol**																																			
	**Bedrails**											**Overbed Table**											**Worktop**													
	Day 1		Day 2		Day 3		Day 4			Day 5		Day 1		Day 2		Day 3		Day 4			Day 5		Day 1		Day 2		Day 3		Day 4			Day 5				
	Bed 3	Bed 4	Bed 3	Bed 4	Bed 3	Bed 4	Bed 4	***Bed 5***	***Bed 6***	Bed 4	Bed 5	Bed 3	Bed 4	Bed 3	Bed 4	Bed 3	Bed 4	Bed 4	***Bed 5***	***Bed 6***	Bed 4	Bed 5	Bed 3	Bed 4	Bed 3	Bed 4	Bed 3	Bed 4	Bed 4	***Bed 5***	***Bed 6***	Bed 4	Bed 5			
t0	0	0	89	27	1	1	4	10	1	0	0	49	60	0	0	56	19	41	40	52	7	95	27	73	4	48	15	25	219	56 #	70	4	31			
t1	3	10	0	1	9	26	45	54 #	151 #	6	7	45	79	5	5	6	22	51	18	56	6	10	2	4	10	10	9	13	1	11	10	17	0			
t2	33	8	82	6	3	23	16	35	15	2	2	3	118	22	46	13	7	15	31	70	23	24	127	19	15	75	1	74	11	44 #	38	28	12			
t3	193	29	25	28	100	45	9	12	16	4	10	247	20	5	12	51	99	29	16	21	15	5	24	10	10	33	37	87	63	25	15 #	13	9			
t4	45	4	24	15	6	19	1	9	1	2	8	5	28	78	55	73	10	15	20	76	19	12	61	8	19	9	51	143	1	66 #	27	40	5			
	**Wipe Protocol**												**Standard Protocol**																							
	**Infusion Pumps**												**Infusion Pumps**																							
	Day 1		Day 2		Day 3		Day 4			Day 5			Day 1		Day 2		Day 3		Day 4			Day 5														
	Bed 1	Bed 2	Bed 1	Bed 2	Bed 1	Bed 2	Bed 1	Bed 2	***Bed 3***	Bed 1	Bed 2	***Bed 3***	Bed 3	Bed 4	Bed 3	Bed 4	Bed 3	Bed 4	Bed 4	***Bed 5***	***Bed 6***	Bed 4	Bed 5													
t0	99	29	2	1	2	3	6	4	0	24	17	8	1	6	0	0	0	1	18	13	23	18	0													
t1	0	58	0	0	0	1	0	6	72	8	13	50	0	1	0	0	1	0	1	0	1	11	1													
t2	17	0	0	2	1	*	2	0	4	0	10	1 #	11	64	1	23	1	9	11	82	34 #	26	1													
t3	10	55	0	0	1	*	3	0	3	0	17	7 #	2	2	0	10	45	4	5	13 #	38	15	2													
t4	47	0	1	1	0	*	2	1	1	0	7	6 #	7	75	4	19	54	83	129	29	135	1	6													

## References

[1] Otter J.A., Yezli S., French G.L. (2011). The Role Played by Contaminated Surfaces in the Transmission of Nosocomial Pathogens. Infect. Control Hosp. Epidemiol..

[2] Otter J.A., Yezli S., Salkeld J.A., French G.L. (2013). Evidence that contaminated surfaces contribute to the transmission of hospital pathogens and an overview of strategies to address contaminated surfaces in hospital settings. Am. J. Infect. Control.

[3] Rutala W.A., Weber D.J., HICPAC (2008). Guideline for Disinfection and Sterilization in Healthcare Facilities, Centers for Disease Control, US.

[4] Donskey C.J. (2013). Does improving surface cleaning and disinfection reduce health care-associated infections?. Am. J. Infect. Control.

[5] Centers for Disease Control and Prevention (2003). Guidelines for environmental infection control in health-care facilities: Recommendations of CDC and the Healthcare Infection Control Practices Advisory Committee (HICPAC). MMWR.

[6] White L.F., Dancer S.J., Robertson C., McDonald J. (2008). Are hygiene standards useful in assessing infection risk?. Am. J. Infect. Control.

[7] Wilson A.P.R., Livermore D.M., Otter J.A., Warren R.E., Jenks P., Enoch D.A., Newsholme W., Oppenheim B., Leanord A., McNulty C. (2016). Prevention and control of multi-drug-resistant Gram-negative bacteria: Recommendations from a Joint Working Party. J. Hosp. Infect..

[8] Dancer S.J. (2014). Controlling Hospital-Acquired Infection: Focus on the Role of the Environment and New Technologies for Decontamination. Clin. Microbiol. Rev..

[9] World Health Organization Guidelines for the Prevention and Control of Carbapenem-Resistant Enterobacteriaceae, Acinetobacter Baumannii and Pseudomonas Aeruginosa in Health Care Facilities. http://www.who.int/infection-prevention/publications/guidelines-cre/en/.

[10] Vickery K., Deva A., Jacombs A., Allan J., Valente P., Gosbell I.B. (2012). Presence of biofilm containing viable multiresistant organisms despite terminal cleaning on clinical surfaces in an intensive care unit. J. Hosp. Infect..

[11] Ramm L., Siani H., Wesgate R., Maillard J.-Y. (2015). Pathogen transfer and high variability in pathogen removal by detergent wipes. Am. J. Infect. Control.

[12] Sattar S.A., Bradley C., Kibbee R., Wesgate R., Wilkinson M.A.C., Sharpe T., Maillard J.Y. (2015). Disinfectant wipes are appropriate to control microbial bioburden from surfaces: Use of a new ASTM standard test protocol to demonstrate efficacy. J. Hosp. Infect..

[13] Kenters N., Huijskens E.G.W., de Wit S.C.J., van Rosmalen J., Voss A. (2017). Effectiveness of cleaning-disinfection wipes and sprays against multidrug-resistant outbreak strains. Am. J. Infect. Control.

[14] Bogusz A., Stewart M., Hunter J., Yip B., Reid D., Robertson C., Dancer S.J. (2013). How quickly do hospital surfaces become contaminated after detergent cleaning?. Healthc Infect..

[15] Attaway H.H., Fairey S., Steed L.L., Salgado C.D., Michels H.T., Schmidt M.G. (2012). Intrinsic bacterial burden associated with intensive care unit hospital beds: Effects of disinfection on population recovery and mitigation of potential infection risk. Am. J. Infect. Control.

[16] Italian Workers Compensation Authority, INAIL LINEE GUIDA SUGLI STANDARD DI SICUREZZA E DI IGIENE NEL REPARTO OPERATORIO. https://www.inail.it/cs/internet/docs/linee-guida-igiene-reparto-operatorio.pdf?section=attivita.

[17] Bannerman T.L., Hancock G.A., Tenover F.C., Miller J.M. (1995). Pulsed-field gel electrophoresis as a replacement for bacteriophage typing of Staphylococcus aureus. J. Clin. Microbiol..

[18] Seifert H., Dolzani L., Bressan R., van der Reijden T., van Strijen B., Stefanik D., Heersma H., Dijkshoorn L. (2005). Standardization and Interlaboratory Reproducibility Assessment of Pulsed-Field Gel Electrophoresis-Generated Fingerprints of Acinetobacter baumannii. J. Clin. Microbiol..

[19] Dancer S.J. (2004). How do we assess hospital cleaning? A proposal for microbiological standards for surface hygiene in hospitals. J. Hosp. Infect..

[20] Wiemken T.L., Curran D.R., Pacholski E.B., Kelley R.R., Abdelfattah R.R., Carrico R.M., Ramirez J.A. (2014). The value of ready-to-use disinfectant wipes: Compliance, employee time, and costs. Am. J. Infect. Control.

[21] Sattar S.A., Maillard J.Y. (2013). The crucial role of wiping in decontamination of high-touch environmental surfaces: Review of current status and directions for the future. Am. J. Infect. Control.

[22] Toffolutti V., Reeves A., McKee M., Stuckler D. (2017). Outsourcing cleaning services increases MRSA incidence: Evidence from 126 English acute trusts. Soc. Sci. Med..

[23] Aldeyab M.A., McElnay J.C., Elshibly S.M., Hughes C.M., McDowell D.A., McMahon M.A.S., Scott M.G., Kearney M.P. (2009). Evaluation of the Efficacy of a Conventional Cleaning Regimen in Removing Methicillin-Resistant Staphylococcus aureus From Contaminated Surfaces in an Intensive Care Unit. Infect. Control Hosp. Epidemiol..

[24] Stewart M., Bogusz A., Hunter J., Devanny I., Yip B., Reid D., Robertson C., Dancer S.J. (2014). Evaluating Use of Neutral Electrolyzed Water for Cleaning Near-Patient Surfaces. Infect. Control Hosp. Epidemiol..

